# The Genomic Potentials of NOB and Comammox *Nitrospira* in River Sediment Are Impacted by Native Freshwater Mussels

**DOI:** 10.3389/fmicb.2018.02061

**Published:** 2018-09-04

**Authors:** Ellen M. Black, Craig L. Just

**Affiliations:** Department of Civil and Environmental Engineering, University of Iowa, Iowa City, IA, United States

**Keywords:** Upper Mississippi River, freshwater mussels, *Nitrospira*, comammox, nitrification, ammonia oxidizing bacteria, nitrite oxidizing bacteria

## Abstract

Freshwater mussel assemblages of the Upper Mississippi River (UMR) sequester tons of ammonia- and urea-based biodeposits each day and aerate sediment through burrowing activities, thus creating a unique niche for nitrogen (N) cycling microorganisms. This study explored how mussels impact the abundance of N-cycling species with an emphasis on *Candidatus* Nitrospira inopinata, the first microorganism known to completely oxidize ammonia (comammox) to nitrate. This study used metagenomic shotgun sequencing of genomic DNA to compare nitrogen cycling species in sediment under a well-established mussel assemblage and in nearby sediment without mussels. Metagenomic reads were aligned to the prokaryotic RefSeq non-redundant protein database using BLASTx, taxonomic binning was performed using the weighted lowest common ancestor algorithm, and protein-coding genes were categorized by metabolic function using the SEED subsystem. Linear discriminant analysis (LDA) effect sizes were used to determine which metagenomes and metabolic features explained the most differences between the mussel habitat sediment and sediment without mussels. Of the N-cycling species deemed differentially abundant, *Nitrospira moscoviensis* and “*Candidatus* Nitrospira inopinata” were responsible for creating a distinctive N-cycling microbiome in the mussel habitat sediment. Further investigation revealed that comammox *Nitrospira* had a large metabolic potential to degrade mussel biodeposits, as evidenced the top ten percent of protein-coding genes including the cytochrome c-type biogenesis protein required for hydroxylamine oxidation, ammonia monooxygenase, and urea decomposition SEED subsystems. Genetic marker analysis of these two *Nitrospira* taxons suggested that *N. moscoviensis* was most impacted by diverse carbon metabolic processes while “*Candidatus* Nitrospira inopinata” was most distinguished by multidrug efflux proteins (AcrB), NiFe hydrogenase (HypF) used in hydrogen oxidation and sulfur reduction coupled reactions, and a heme chaperone (CcmE). Furthermore, our research suggests that comammox and NOB *Nitrospira* likely coexisted by utilizing mixotrophic metabolisms. For example, “*Candidatus* Nitrospira inopinata” had the largest potentials for ammonia oxidation, nitrite reduction with NirK, and hydrogen oxidation, while NOB *Nitrospira* had the greatest potential for nitrite oxidation, and nitrate reduction possibly coupled with formate oxidation. Overall, our results suggest that this mussel habitat sediment harbors a niche for NOB and comammox *Nitrospira*, and ultimately impacts N-cycling in backwaters of the UMR.

## Introduction

Water quality of the Upper Mississippi River (UMR) has been documented for decades (Lerch et al., 2015), yet the UMR basin contributes over 50,000 metric tons of bioactive nitrogen (N) to the Gulf of Mexico each year (Donner and Kucharik, 2008). Research has shown that microbial communities are impacted by the addition of bioactive N (Hallin et al., 2009), and subsequently alter N-biogeochemical cycling in the UMR through nitrification and denitrification processes (Mulholland et al., 2008; Shange et al., 2012). Enhancing the vertical exchange between overlying water and groundwater (i.e., water-sediment interface) of UMR backwater channels has been proposed to significantly enhance N removal (Strauss et al., 2006; Gomez-Velez et al., 2015), particularly because biotic removal of N reaches a maximum efficiency of 40% as N loads increase in large streams (Mulholland et al., 2008) and denitrification rates plateau as nitrate (NO_3_-N) reaches 5 mg/L in backwater channels (Kreiling et al., 2011). Taken together, these findings emphasize the large N-cycling potential of benthic organisms, by enhancing the flux of nutrients into sediment for microbial transformations (Vaughn and Hakenkamp, 2001; Atkinson et al., 2013).

Freshwater mussels (order Unionidae) native to the UMR live in assemblages of 3–5 mussels m^-2^, collectively filter billions of gallons of water, and remove tons of N-containing biomass from overlying water each day (Newton et al., 2011). In addition to the ecosystem services of water filtration and enhancing nutrient exchange rates across the water-sediment interface (Atkinson et al., 2014), mussel excretion of feces and pseudofeces (biodeposition products) sequesters ammonia (NH_3_) and carbon (C) into sediment porewater (Newton et al., 2011; Bril et al., 2014, 2017). As a result, mussel assemblages are attributed with creating “hotspots” of N and C in surrounding sediment (Atkinson and Vaughn, 2015), and create a microbial niche ripe for nitrification at the interface of oxic and anoxic conditions (Black et al., 2017).

Nitrifying organisms capable of mixotrophy may pose an advantage in a mussel-influenced habitat, owing to the adaptation of switching metabolic functions when conditions change from oxic to anoxic (Matantseva and Skarlato, 2013; Daebeler et al., 2014). It was previously thought that nitrite (${\text{NO}}_{2}^{-}$) oxidizing bacteria (NOB) were restricted to oxic environments where ammonia (NH_3_) oxidizing bacteria (AOB) produce ${\text{NO}}_{2}^{-}$, but recent genomic analyses have expanded the known metabolic functions of conventional NOB *Nitrospira*. For example, the ${\text{NO}}_{2}^{-}$ oxidizing species *Nitrospira moscoviensis* is genetically capable of cyanate degradation (Palatinszky et al., 2015), aerobic hydrogen oxidation (Koch et al., 2014), and formate oxidation coupled with nitrate (${\text{NO}}_{3}^{-}$) reduction (Koch et al., 2015). Nitrification was further expanded after discovering that *Nitrospira moscoviensis* can produce NH_3_ and CO_2_ by way of urea hydrolysis, and can reciprocally feed NH_3_ to urease-lacking AOB and receive ${\text{NO}}_{2}^{-}$ in return (Koch et al., 2015). Furthermore, the N-cycle was transformed after the discovery of a single organism capable of complete NH_3_ oxidation (comammox) (van Kessel et al., 2015) and confirmation of genes required for complete nitrification encoded by “*Candidatus* Nitrospira inopinata” (Daims et al., 2015), and potentially even sulfur reduction (Camejo et al., 2017).

In a previous study, we showed that sediment of a well-established mussel habitat in UMR backwaters contained an enhanced niche for Nitrospirae in addition to a greater abundance of microorganisms indicative of an oxic-anoxic niche, like anaerobic ammonium oxidizers (anammox). This presumed oxic-anoxic niche was detected closer to the water-sediment interface in the mussel habitat, since the relative abundance of anammox bacteria peaked at shallow (3 cm) sediment depths with mussels, but were more abundant in deeper (5 cm) sediments in the no-mussel treatment (Black et al., 2017). Furthermore, the 16S rRNA amplicon survey showed fewer differences among N-cycling phylotypes in shallow sediment with mussels and deeper sediment without mussels (i.e., intrasample differences), and the fewest differences when comparing the shallow mussel sediment against the deeper no-mussel sediment (inter-sample differences). In response, this study used the deeper no-mussel sediment as the most stringent baseline to assess mussel influences on the N-cycling community with mussels. We employed metagenomic shotgun sequencing of total DNA corresponding with the aforementioned oxic-anoxic niche sediment components, with the goal of identifying the N-cycling species most impacted by mussels. We hypothesized that sediment from the mussel habitat would contain an increased abundance of nitrifying taxons, presumably due to an enhanced genomic potential for ammonia oxidation.

## Materials and Methods

### Sediment Collection and DNA Isolation

Our study sites were located in the backwaters of the UMR navigation pool 16, where a consistently populated mussel assemblage has been studied for decades (USACE, 1981, 1984; Young et al., 2005; Morales et al., 2006). Sediment cores were obtained from the mussel habitat (41.452804, -90.763299) and upstream sediment (41.451540, -90.753275) lacking mussels (Black et al., 2017); both sites had similar hydraulics and sediment composition (Young et al., 2005), and will be considered as treatments “with-mussels” and with “no-mussels” according to previous studies (Mirto et al., 2000; Danovaro et al., 2004). Cores were removed from each site using a 2-in diameter, post-driver with a polypropylene liner (Multi-State Sediment Sampler, Art’s Manufacturing and Supply, Inc.; American Falls, ID, United States), and an ethanol flame-sterilized 3/8-in diameter drill bit was used to penetrate the cores at depths of 3 and 5 cm. For each core, samples (0.25 g sediment) were removed in quadruplicate (*n* = 4, 3 cm depth with mussels; *n* = 4, 5 cm depth without mussels) and stored in sterile bead beating tubes overnight at -20^o^C. Genomic DNA was isolated (PowerSoil DNA Isolation Kit; MoBio Laboratories, Inc., Carlsbad, CA, United States) and stored at -20°C. Following verification of DNA quality and quantity (NanoDrop 1000; Thermo Fisher Scientific, Waltham, MA, United States), genomic DNA was sequenced at the University of Iowa Institute for Human Genetics (IIHG). As mentioned earlier, selection of these samples were informed by evidence suggesting oxic-anoxic interface niches were located at 5 cm sediment depth without mussels and 3 cm depth beneath mussels (Black et al., 2017). Samples chosen for this experiment correspond to the following 16S rRNA amplicon sequencing data at MG-RAST: without-mussels- mgm4705698.3, mgm4705704.3, mgm4705686.3, mgm4705697.3 with-mussels mgm4705708.3, mgm4705672.3, mgm4705699.3, mgm4705680.3.

### Metagenomic Shotgun Sequencing

For each sample, 120 ng of genomic DNA in 60 μL of 10 mM Tris-HCl, pH 8.0 buffer, was placed into 1.5 mL RNase-/DNase-free, low binding microcentrifuge tubes. Library creation steps were performed by the IIHG Genomics Division, and included DNA shearing using the Covaris Adaptive Focused Acoustics^TM^ process (Covaris E220 Focused-ultrasonicator; Covaris, Inc., Woburn, MA, United States), and DNA fragment purification and end polishing (KAPA Hyper prep kits; Kapa Biosystems, Inc., Wilmington, MA, United States) prior to ligation to indexed adaptors. The library size distribution was validated using the Agilent 2100 Bioanalyzer Instrument (Agilent Technologies, Santa Clara, CA, United States), and quantified using the q-PCR KAPA library amplification module following manufacturer instructions (Kapa Biosystems, Inc.). The indexed libraries were normalized, pooled, and clustered on a flow cell using the cBOT Cluster Generation System (Illumina, Inc., San Diego, CA, United States) and sequenced on the Illumina HiSeq 4000 System (Illumina, Inc.) in high output mode (1 lane, 2 × 150 bp). FASTQ files are accessible at ENA (Study Accession: PRJEB23134) and NCBI repositories (BioProject ID: PRJNA414922), and MG-RAST contains QA/QC and analyses of metagenomes (MG-RAST project mgp21252).

### Bioinformatics Pipeline

FastQC (Andrews, 2010) was used for quality control and revealed an average sequence abundance of 42,606,252± 2,365,531 sequences, sequence lengths of 151 bp, and 60% (±0.84%) GC content for no-mussel samples, and 41,402,435 ± 3,444,191 sequences, with sequence lengths of 151 bp, and 60% (±1.31%) GC content for samples with mussels. For taxonomic and functional binning of reads, we employed the streamlined DIAMOND (Buchfink et al., 2015) and MEGAN (Huson et al., 2007) pipeline specialized for microbiome shotgun sequencing analyses (Huson et al., 2016). First, RefSeq (O’Leary et al., 2016) non-redundant (nr) archaeal and bacterial protein sequences (Release80) were concatenated to construct a database for BLASTx alignments in DIAMOND using the “make.db” command, and pairwise alignments were performed using the default BLASTx settings (BLOSUM62 matrix, γ = 0.267, *K* = 0.041, Penalties = 11/1). The aligned reads from DIAMOND were imported into MEGAN using *daa-meganizer* (Huson et al., 2016), keeping only the top 100 matches per read. The weighted lowest common ancestor (LCA) algorithm was used for taxonomic binning using default settings in MEGAN6 (Huson et al., 2016) (min score = 50.0, max expected = 0.01, top percent = 10.0%, min support percent = 0.05, min support = 1, 80% coverage for weighted LCA algorithm) and classified according to NCBI taxonomy IDs (Nov 2016 release). Furthermore, aligned reads were assigned functional roles using accession mapping files for SEED subsystems (Overbeek et al., 2005) (SEED May 2015 annotation). The resulting files contained all reads, alignments, taxonomic and functional classifications, and were normalized for sampling read depth (normalized to 12,726,950 reads per sample) and assigned metadata categories, “no-mussel” and “with-mussel.” Reads aligned to N-cycling genomes were extracted from MEGAN for differential abundance analysis using LefSe (Segata et al., 2011). The most differentially abundant genomes belonged to “*Candidatus* Nitrospira inopinata” (GCF_001458695.1) and *Nitrospira moscoviensis* (GCF_001273775.1), and were further assembled and annotated in Unipro UGENE (Okonechnikov et al., 2012) and depicted using DNAPlotter (Carver et al., 2009). The UGENE workflow included mapping reads to indexed reference genomes using BWA MEM (Li and Durbin, 2009) with default settings, followed by filtering and sorting the BAM files using SAMtools (Li et al., 2009), and a final quality control step using FastQC (Andrews, 2010).

### LDA Effect Size

A linear discriminant analysis (LDA) method was used to assess which genomes and genomic features were most discriminative of the freshwater mussel habitat. N-cycling taxonomies of interest were chosen based on previous research (Pfister et al., 2014) and included AOB and NOB phylotypes with the prefix “nitro,” N-reducing phylotypes designated by “denitrificans” or “nitroreducens,” and anammox candidate genera, *Brocadia* and *Jettenia*. The relative abundance of reads aligned to these N-cycling taxons were assessed for LDA effect size (LEfSe) (Segata et al., 2011) to determine which N-cycling taxonomic features were most responsible for differences in the mussel habitat microbiomes. All samples were labeled by class (“mussel” and “no-mussel”) and features were compared for differential distribution using the non-parametric factorial Kruskal–Wallis sum-rank test (α = 0.05) and normalized to a total read count of 1 M. Features deemed differentially abundant were compared for significant effect size using the pairwise Wilcoxon rank-sum test (α = 0.05; “all against all”), and ranked features according to greatest effect size. A minimum LDA score of 2.0 was chosen as a cutoff for significant features to limit analysis to the most distinctive metagenomic traits. “*Candidatus* Nitrospira inopinata” and *Nitrospira moscoviensis* were shown to be the most distinctive genomes with mussels, and follow-up genetic marker tests were performed for SEED assignments to address which metabolic functions may be responsible for this differentiation.

## Results

### N-cycle Taxonomic Composition

**FIGURE 1:**
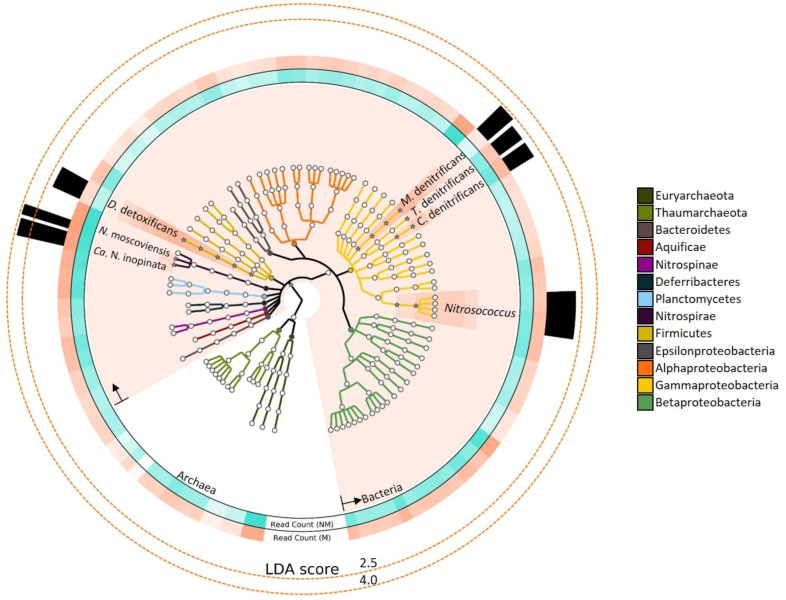
Nitrogen-cycling taxonomies assessed for linear discriminant analysis (LDA) effect size are colored based on phylum, as specified in the legend. Taxons shown with a “^∗^” icon and salmon-colored background had statistically significant LDA scores (LDA > 2, *P* < 0.05). Rings surrounding the phylogenetic tree depict the relative abundance of reads assigned to the respective species. Ring color intensity represents relative read count with mussels (salmon-colored; “M”) and with no mussels (turquoise-colored; “NM”). The opacity of “Read count” ring-segments corresponds to the greatest taxonomic abundance. Several N-cycling taxons were differentially abundant with mussels and LDA effect sizes are represented by the height of black bars in the outer-most ring.

The DIAMOND/MEGAN pipeline revealed metagenomic reads assigned to N-cycling organisms (**Figure 1**) were slightly more abundant with mussels (157,275 ± 17,503 reads) than without mussels (136,884 ± 20,982 reads), and the mussel habitat contained more reads belonging to N-cycling bacterial lineages (**Figure 1**) with Nitrospirae representing the most bacterial species. In both treatments, “*Candidatus* Methanoperdens nitroreducens” represented the largest number of archaeal metagenomic reads but was not differentially abundant between treatments. ${\text{NO}}_{2}^{-}$ oxidizing organisms represented the largest N-cycling group, with *Nitrospira moscoviensis*, *Nitrospira defluvii*, and “*Candidatus* Nitrospira inopinata” comprising an average of 22, 15, and 11% of N-cycling metagenomic reads in the mussel habitat, respectively. *Steroidobacter denitrificans* was also a highly abundant component of the N-cycling community (12–14%) but was not differentially abundant between treatments.

Linear discriminant analysis effect size of the metagenomic reads assigned to N-cycle taxons further emphasized the major differences in the mussel habitat (**Figure 1**). Of the taxons considered, bacterial lineages experienced the most increases with mussels (LDA = 4.27, *P* = 0.043), and the most differentially abundant species were *Nitrospira moscoviensis* (LDA = 3.80, *P* = 0.021) and “*Candidatus* Nitrospira inopinata” (LDA = 3.63, *P* = 0.021). One other group of nitrifying taxons were differentially greater with mussels, and belonged to the *Nitrosococcus* genus (LDA = 2.20, *P* = 0.021). Multiple denitrifying taxons were greater with mussels, including *Methylomonas denitrificans*, *Denitrobacterium detoxificans*, *Competibacter denitrificans*, and a Gammaproteobacterial sulfur oxidizing symbiont, *Thiohalorhabdus denitrificans*. However, these denitrifying species were lower in abundance than *Nitrospira*, and thus were ranked lower as biomarker species. Protein functional assignments of “*Candidatus* Nitrospira inopinata” and *N*. *moscoviensis* were also assessed for distinctive features between the mussel and no-mussel treatments, with the goal of discovering niche differentiating functions responsible for the enhanced abundance of NOB and comammox *Nitrospira* genomes (McGill et al., 2007).

### *Nitrospira moscoviensis* Genomic Potential

A total of 435,151 SEED protein functions were assigned to the genome of *N. moscoviensis* (normalized to 36,562 per sample) and was dominated by 5 SEED categories: carbohydrates, cofactors, vitamins, prosthetic groups, pigments, amino acids and derivatives, protein metabolism, and DNA metabolism (**Supplementary Table S1**). The 25% most abundant SEED subsystems included those indicative of metabolic activity and growth, such as peptidoglycan and cytoskeleton biosynthesis, respiration and carbon fixation, DNA replication and repair (**Supplementary Table S1**). Highly abundant SEED proteins unique to *N. moscoviensis* in the no-mussel treatment included those potentially functioned in motility, chemotaxis, biotin synthesis, and thiamin metabolism (“5-FCL-like protein”), while the mussel habitat treatment was dominated by genes encoding folate and cysteine biosynthesis, carbon cycling (“alpha carboxysome”), and the DNA regulatory proteins YebC and proteasomes (**Supplementary Table S1**).

Of the N-metabolism functional assignments (**Table 1**), formate hydrogenases were the most abundant N-cycling category for both treatments, and numerous enzymatic functions were relatively more abundant in the mussel habitat treatment. These included genes encoding an ${\text{NH}}_{4}^{+}$ permease, NO reductase proteins (NorD and NorQ), formate dehydrogenase subunits (beta and chain D), ${\text{NO}}_{2}^{-}$/${\text{NO}}_{3}^{-}$ transporters and sensors, periplasmic nitrate reductases (NapG and NapF), and urease proteins (UreA and ureF) (**Table 1**).

**Table 1:** *Nitrospira moscoviensis* N-cycling protein functions from the mussel habitat in relative abundance (RPKM) and as a proportion of SEED enzymatic function.

SEED subsystems and protein functions	Average read abundance (RPKM)	Relative proportion of protein function
**Ammonia assimilation**		
Nitrogen regulatory protein P-II Gln	1409.04	0.34%
Nitrogen assimilation regulatory protein Ntr	395.687	
Ammonium transporter AmtB	300.22	
Glutamate-ammonia-ligase GlnEb	3267.89	
**Denitrification**		
Ferredoxin-type protein NapG (periplasmic nitrate reductase)	801.88	0.13%
Copper-containing nitrite reductase NirK (NO-forming)	ND	
**Formate hydrogenase**		
Formate hydrogenlyase, membrane subunit HyfB	95.37	0.71%
Putative formate hydrogenlyase, membrane subunit HyfC	ND	
Putative formate hydrogenlyase, membrane subunit HyfE	214.91	
Formate hydrogenlyase, membrane subunit HyfF	158.95	
Formate hydrogenlyase, large subunit HyfG	ND	
Putative formate hydrogenlyase, small subunit HyfI	94.81	
Formate dehydrogenase, alpha subunit FdsA	17.577	
Formate dehydrogenase, beta subunit FdsB	60.937	
Formate dehydrogenase, gamma subunit FdsG	ND	
Formate hydrogenlyase transcriptional activator FhlA	122.83	
Formate transporter FocA	ND	
**Nitrate and nitrite ammonification**		
Nitrate transporter NarK	80.79	0.15%
Nitrate ABC transporter Nrt	333.27	
Nitrite oxidoreductase, alpha subunit NxrA	1097.03	
Nitrite oxidoreductase, beta subunit NxrB	ND	
Nitrite oxidoreductase, membrane subunit NxrC	1695.47	
Nitrite reductase (NADH) small subunit NirD	474.06	
**Nitrogen fixation**		
Nitrogenase (molybdenum-iron)-specific transcriptional regulator NifA	63.33	0.15%
Nitrogenase (iron-iron) transcriptional regulator	ND	
**Urea degradation**		
Urease alpha subunit UreC	139.91	0.10%
Urease gamma subunit UreA	159.583	
Urea ABC transporter, urea binding protein UrtA	36.72	
Urease accessory protein UreD	ND	
Urease accessory protein UreF	70.38	
Urease accessory protein UreG	143.91	
Urease beta subunit UreB	ND	

*SEED counts are shown as a percentage of the total classified protein function in the mussel habitat.*

#### *N. moscoviensis* Genetic Markers

**FIGURE 2:**
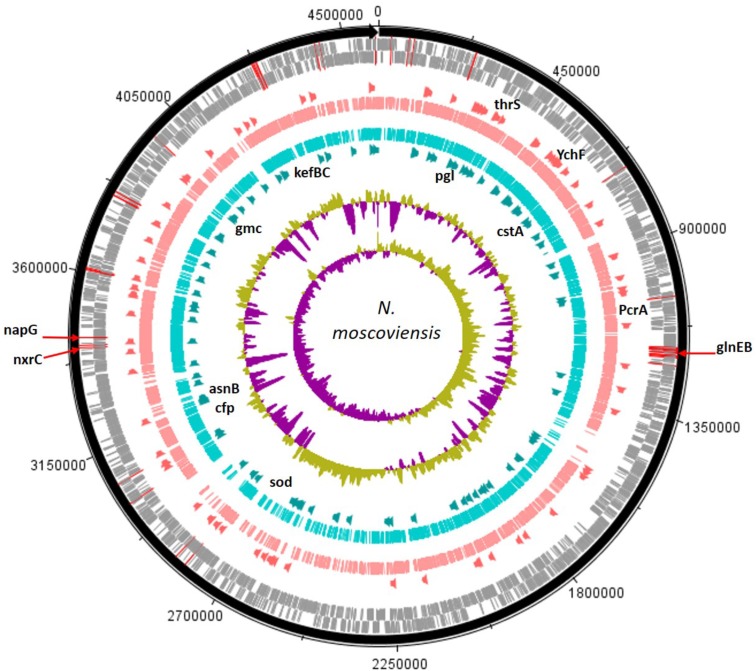
The assembled *N. moscoviensis* genome is depicted with tick mark intervals of 225 kBp, and tracks are composed of the following components, starting with the outermost rings: Forward strand coding regions (gray), reverse strand coding regions (gray), the top 25% most abundant enzymes with mussels (dark orange), genomic read coverage from the mussel habitat (salmon colored), genomic coverage without mussels (turquoise colored), the top 25% most abundant enzymes with no mussels (dark green), GC coverage, and GC skew. The N-cycling genes with greatest abundance, GlnEB (NITMOv2_1289, NITMOv2_1290), nitrite oxidoreductase C (NITMOv2_3624), and periplasmic nitrate reductase (NITMOv2_3626) are marked with red lines over the outer rings. Gene names are shown for those with the largest LDA effect size (LDA > 3.0). For the mussel habitat, these include the YebC-like proteins, YchF and thrS, heme utilization protein, and DNA helicase (PcrA). Without mussels, these include the CPA2 protein families (kefBC in *N. moscoviensis*), 6-phosphogluconolactonase (pgl), a Fe-Mn superoxide dismutase (sod), asparagine synthetase (asnB), glucose methanol choline oxidoreductase (gmc), pyruvate decarboxylase (cfp), and carbon starvation protein (cstA).

Genetic code processing was a major potential function of *N*. *moscoviensis* in the mussel habitat, as evidenced by increased functional proteins used in DNA, RNA, and protein metabolism (LDA up to 3.32). Two of the most distinct features were genes encoding YebC-like DNA-binding regulatory proteins, an ATP-dependent DNA helicase protein (PcrA), and ribonuclease H III (**Supplementary Table S2** and **Figure 2**), in addition to other proteins like DNA polymerase III and LSU ribosome. Unclassified hypothetical proteins (FIG039061) related to heme utilization was a highly ranked SEED subsystem (LDA = 3.33, *P* = 0.043) and likely was due to a large abundance a gene encoding a modular heme utilizing protein (NITMOv2_0147), with other iron-based genetic markers including Cytochrome C553 and an Fe-S cluster regulator (IscR). Other differentially abundant features were related to carbon cycling SEED subsystems (LDA = 2.99, *P* = 0.021) and included genes encoding ribulose phosphate-3 epimerase used for carbon fixation, and two glycogen synthesis enzymes, 4-alpha-glucanotransferase (MalQ), and 1,4-alpha glucan (glycogen) branching enzyme (GH-13 type) (GlgB).

Contrastingly, the *N. moscoviensis* potential protein functions without mussels were largely marked by genetic markers of stress response. The glutathione-regulated K^+^ efflux system used in potassium metabolic processes (LDA = 3.28, *P* = 0.043) were the most definitive SEED subsystems of the no-mussel *N. moscoviensis* genome. Furthermore, the glutathione-regulated K^+^ efflux system protein family (KefB) is activated in the presence of methylglyoxal (Ferguson et al., 1995), and the metabolism of methylglyoxal was also a genetic marker of the no-mussel treatment (LDA = 2.45, *P* = 0.043). These results may suggest a stress response genetic marker, as glutathione-regulated potassium efflux systems are often utilized to counteract electrophilic compounds during stress (Tötemeyer et al., 1998; Bott and Love, 2004). Additionally, superoxide dismutase was another highly ranked functional marker and may suggest an enhanced stress from reactive oxygen. Other stress genetic markers included the carbon starvation stress SEED subsystem (LDA = 2.85, *P* = 0.021) and an enhanced abundance of genes encoding carbon starvation protein A (CstA) (**Supplementary Table S2**). Other genetic markers suggest a potential metabolic ability to respond to diverse carbon compounds, including genes encoding gluconolactonase of the Entner Duodoroff pathway (LDA = 2.99, *P* = 0.021), acetoacetate metabolism with an enhanced regulatory protein, AtoC, and an acetyl-CoA biosynthesis enzyme, pyruvate decarboxylase (**Supplementary Table S2**). Differential features also included the potential metabolism of complex carbon sources, such as lactose (LDA = 2.40, *P* = 0.021) via 2-oxoglutarate decarboxylase, mannose by way of mannose-1-P guanylyltransferase, maltose and maltodextrin degradation via alpha amylase, and *N*-acetylglucosamine (LDA = 2.96, *P* = 0.043) with beta-hexosaminidase enzymes.

N metabolic genes were lowly abundant for *N. moscoviensis* genomes in both treatments, but a ${\text{NO}}_{3}^{-}$/${\text{NO}}_{2}^{-}$ sensor protein was a functional marker of the mussel habitat (LDA = 2.61, *P* = 0.014). This enhanced ability to transport ${\text{NO}}_{2}^{-}$ and ${\text{NO}}_{3}^{-}$ across the cell membrane could be linked to potential energy conservation processes or metabolic reactions beyond N-cycling. For example, the Nxr protein of *N. moscoviensis* is contained within the periplasm and does not require transportation of ${\text{NO}}_{2}^{-}$ or ${\text{NO}}_{3}^{-}$ into the cell (Spieck et al., 1998). Instead, *N. moscoviensis* likely transports ${\text{NO}}_{2}^{-}$ and ${\text{NO}}_{3}^{-}$ into the cell for assimilation and outside the cell to protect against excess ${\text{NO}}_{2}^{-}$ (Lücker et al., 2010).

In comparison, the no-mussel treatment showed an enhance genetic ability to uptake and store N by way of encoding a cyanate ABC transporter (LDA = 2.40, *P* = 0.043) and asparagine synthetase (AsnB) (**Supplementary Table S2**), respectively. Differential abundance of cyanate transporters would indicate *N. moscoviensis* could have increased ability to obtain an alternative source of N via the cyanase enzyme (Koch et al., 2015) or could be reciprocally fed to cyanase-lacking nitrifiers (Palatinszky et al., 2015). The co-increases of genes encoding AsnB and a cyanate transporter suggests that *N. moscoviensis* may have utilized alternative N sources in the sediment without mussels, perhaps due to inorganic N limitations in its environment.

### Comammox *Nitrospira* Genomic Potential

**FIGURE 3:**
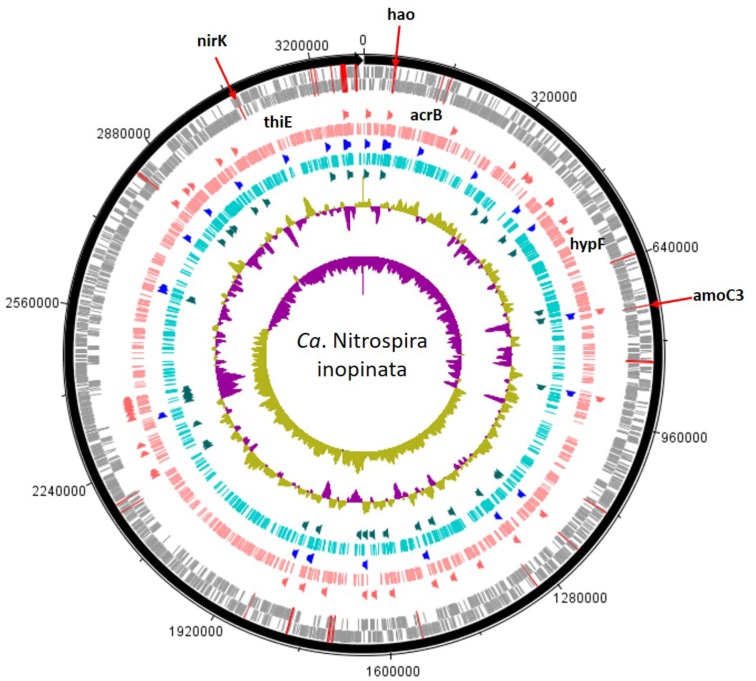
*Candidatus* Nitrospira inopinata assembly shown with tick mark intervals of 160 kBp. Tracks within the genome are composed of the following, starting with the outermost rings: Forward strand coding regions, reverse strand coding regions, the top 25% most abundant enzymes with mussels (dark orange), genomic coverage with mussels (salmon colored), top 25% most abundant SEED subsystems in both treatments (royal blue), genomic coverage without mussels (turquoise colored), the top 25% most abundant enzymes with no mussels (dark green), GC coverage, and GC skew. N-cycling genes are designated with red lines across the CDS rings and the most abundant genes [amoC3 (NITINOP_0766), nirK (NITINOP_3146), hao (NITINOP_0065)] are designated with text. For simplicity, genes with LDA > 3 are named (acrB, hypF, and thiE), and therefore limits to the mussel habitat treatment (text shown above salmon-colored ring).

A total of 163,253 SEED functionalities were assigned to the genome of “*Candidatus* Nitrospira inopinata” (normalized to 13,278 per sample) and the top 25% most prominent SEED subsystems did not differ substantially between treatments (**Supplementary Table S3** and **Figure 3**). The “restriction-modification system” represented the largest potential functional category for the no-mussel treatment, while the potential for thiamin metabolism (“5-FCL-like protein”), and stress response (“commensurate regulon activation”) SEED assignments were the most abundant for the genome with mussels. The metabolic potential for “urea decomposition” was more abundant without mussels, while “biogenesis of c-type cytochromes” was greater in the mussel treatment. The “ammonia monooxygenase” potential function was the 4^th^ most abundant SEED subsystem for both mussel and no-mussel treatments. Both treatments shared numerous abundant SEED assignments (**Supplementary Table S3**), including genes encoding a resistance-nodulation-cell division (RND) efflux system inner membrane transporter, AmoC, alcohol dehydrogenase, and glycogen utilizing enzymes (**Supplementary Table S3**). Numerous N-cycling functions of “*Candidatus* Nitrospira inopinata” were highly abundant in the mussel habitat (**Table 2**), including genes encoding NO- and N_2_O-forming enzymes, Amo, and ${\text{NO}}_{2}^{-}$/${\text{NO}}_{3}^{-}$ transforming enzymes (Nxr, NapG, and Nas). Although the total abundance of genes encoding Amo was slightly larger in the mussel treatment, this trend was most evident for AmoA and AmoB. Genes encoding urea transporters (UrtB and UrtC) and urease proteins (UreD, UreG, UreA, and UreF) were relatively more abundant in the mussel treatment, though the “urea decomposition” subsystem did not follow this overall trend.

**Table 2:** “*Candidatus* Nitrospira inopinata” N-cycling protein functions from the mussel habitat as relative abundance (RPKM) and as a proportion of SEED enzymatic function.

SEED subsystems and protein functions	Average read abundance (RPKM)	Relative proportion of protein function
**Ammonia monooxygenase**		
Ammonia monooxygenase A-subunit AmoA	2955.70	3.79%
Ammonia monooxygenase B-subunit AmoB	1405.72	
Ammonia monooxygenase C-subunit AmoC	37945.15	
**Urea decomposition**		
Urea ABC transporter, ATPase protein UrtD	1634.33	3.52%
Urea ABC transporter, ATPase protein UrtE	1459.22	
Urea ABC transporter, permease protein UrtB	1713.41	
Urea ABC transporter, permease protein UrtC	735.02	
Urea ABC transporter, urea binding protein UrtA	1443.89	
Urea carboxylase-related amino acid permease UctT	201.53	
Urease accessory protein UreD	ND	
Urease accessory protein UreF	151.66	
Urease accessory protein UreG	303.31	
Urease alpha subunit UreC	724.79	
Urease beta subunit UreB	221.21	
Urease gamma subunit UreA	ND	
**Denitrification**		
Copper-containing nitrite reductase NirK	10156.73	0.50%
Nitric oxide reductase protein NorQ	127.68	
**Nitrate and nitrite ammonification**		
Hydroxylamine dehydrogenase (Hao)	3472.95	0.28%
Putative HaoB	107.52	
Nitrite/Nitrate oxidoreductase, alpha subunit (NxrA)	ND	
Nitrite/Nitrate oxidoreductase, beta subunit (NxrB)	ND	
putative Nitrite/Nitrate oxidoreductase, membrane subunit (NxrC)	522.64	
**Ammonia assimilation**		
Ammonium transporter Rh50	87.70	0.17%
Glutamate-ammonia-ligase (GlnEb)	271.58	

*SEED counts are shown as a percentage of the total classified protein function in the mussel habitat.*

Finally, N-cycling genes shared by *N. moscoviensis* and “*Candidatus* Nitrospira inopinata” were assessed for differential abundance to reveal if N-cycling genes could explain how both species were distinct to the mussel habitat, despite competing for similar N substrates. The protein functions present in both species include ${\text{NH}}_{4}^{+}$ transporters and permeases, copper-containing nitrite reductase (NirK), a “NnrS protein involved in response to NO,” NO reductase, periplasmic ${\text{NO}}_{3}^{-}$ reductase, ${\text{NO}}_{2}^{-}$/${\text{NO}}_{3}^{-}$ sensor and response regulator proteins, Nxr, urea transporters, and urease enzymes. A mean rank multiple comparison analysis (Kruskal–Wallis test with Dunn’s multiple test correction) revealed that the sum of genes encoding urea transporters (Urt) (Mean rank difference = 53.8, *P* = 0.002) and copper-containing nitrite reductases (NO-forming) (NirK) (Mean rank difference = 54.0, *P* = 0.002) were more abundant from the “*Candidatus* Nitrospira inopinata” genome (mussel habitat) compared to *N. moscoviensis.* It should be noted that other studies have detected nirK in *Nitrospira* but we are not aware of studies detecting NO production (Camejo et al., 2017). Additionally, it has been suggested that nirK from AOB could catalyze the oxidation of NO to ${\text{NO}}_{2}^{-}$ (Kuypers et al., 2018), but we cannot make conclusions on enzymatic activity from metagenomic evidence alone.

#### Comammox *Nitrospira* Genetic Markers

The most discerning differences in the genome of “*Candidatus* Nitrospira inopinata” with mussels included a gene encoding the RND efflux system inner membrane transporter (AcrB) (**Supplementary Table S4** and **Figure 3**) as part of the SEED subclass “FOL commensurate regulon activation” (LDA = 3.71, *P* = 0.043). Numerous other potential stress response and virulence pathways were greater in the mussel treatment (**Supplementary Table S4**), such as genes encoding a RND efflux system membrane fusion protein (TtgA), a membrane protease with a mechanism for aminoglycoside resistance (HflK), an integral inner membrane protein type IV secretion complex (VirB), and “death on curing protein” (Doc) as part of the pathway of YdcE and YdcD toxin programmed cell death (LDA = 2.46, *P* = 0.014). Results also indicated a larger metabolic potential for metal tolerance in the mussel treatment, namely copper tolerance (LDA = 2.26, *P* = 0.047) by way of a gene encoding a periplasmic divalent cation tolerance protein (CutA).

Other genetic markers in the mussel habitat had indirect links to N metabolism through potential for urea cycling and cytochrome biosynthesis. A gene encoding a NiFe hydrogenase (HypF) assembly protein responsible for regulating the sulfur-reducing hydrogenase gene set (*hydBGDA* and *hybD*) was greater with mussels, and was recently suggested as giving comammox *Nitrospira* the potential function of hydrogen oxidation coupled to sulfur reduction in anaerobic conditions (Camejo et al., 2017). Genes encoding a cytochrome c-type biogenesis protein (CcmF) were greater with mussels, and is a heme chaperone required for biogenesis of cytochrome c-type proteins located immediately downstream of hydroxylamine dehydrogenase (*hao*) (**Figure 3**). Another genetic functional marker greater with mussels encodes carbamoyl phosphate synthetase (CarB) and would effectively add urea-derived NH_3_ into central metabolism.

SEED subsystems of “*Candidatus* Nitrospira inopinata” most indicative of the no-mussel treatment were those potentially functioned in potassium homeostasis (LDA = 3.53, *P* = 0.043), alpha carboxysome (LDA = 3.04, *P* = 0.043), and DNA metabolic CRISPR function (LDA = 3.04, *P* = 0.043). The only significantly different protein-coding gene classified in the “potassium homeostasis” subsystem encoded a potassium transporting ATPase (KdpC), and is a catalytic chaperone for high-affinity potassium uptake (Irzik et al., 2011). Greater function potential for rubrerythrin, a stress response protein used to combat oxidative stress (LDA = 2.45, *P* = 0.021) (Cardenas et al., 2016), was greater in the no-mussel treatment, and may be linked to greater abundances of genes encoding a recombination and repair protein (RecO) and excinuclease ABC genes in DNA repair pathways (LDA = 2.38, *P* = 0.021). N-cycling genes were not found to be a significant feature differentiating comammox *Nitrospira* genomes in the no-mussel treatment.

## Discussion

In support of our research goal, we provided metagenomic evidence of niche partitioning features to explain how two species of *Nitrospira* were greater in UMR sediment with mussels. This aligns with our previous 16S rRNA amplicon sequencing study which detected a 10% greater abundance of Nitrospirae in the mussel habitat (Black et al., 2017). Furthermore, this metagenomic research showed greater abundances of species capable of urea hydrolysis and clarifies our previous assumptions that an alternative source of N allowed co-increases in anammox, AOB, and NOB phylotypes. We found that *Nitrospira moscoviensis* and “*Candidatus* Nitrospira inopinata” were the most differentiating N-cycling taxons in this mussel habitat niche, and these organisms likely co-existed because of metabolic flexibility beyond the conventional N-cycle.

### *Nitrospira moscoviensis* Marked by Genetic Diversity and Potential for Carbon Metabolism

The most definitive genetic marker of *N. moscoviensis* from the mussel habitat encoded YebC (LDA = 3.49) and may suggest the potential for enhanced genetic diversity. For example, the YebC protein regulates genetic recombination and resolution of Holliday Junctions (Zhang et al., 2012), and enzymes classified within the YebC subsystem, YchF and ThrRS, function as translational control factors (Teplyakov et al., 2003; Zhou et al., 2016). These results suggest that *N. moscoviensis* in the mussel habitat had the genetic potential to synthesize proteins and respond to environmental variations (Hershey et al., 2012). Additionally, it makes sense that enhanced DNA repair using “UvrD-related helicases” and “two cell division clusters relating to chromosome partitioning” were also top differentiating features in the mussel habitat. Increased DNA repair and recombination genetic markers would also have permitted *N. moscoviensis* to respond to changing environments by way of genetic diversity. To this point, flexible metabolism and a mixotrophic lifestyle enables *N. moscoviensis* to be ecologically successful in numerous other environments (Koch et al., 2015).

We demonstrated that gene abundances associated with carbon metabolic processes of *N. moscoviensis* were impacted in the mussel habitat, as evidenced by increased Calvin cycle and glycogen metabolism genetic markers while the no-mussel treatments contained carbon stress protein genetic markers. CstA is a membrane protein predicted to be involved in peptide uptake when carbon availability is low (Rasmussen et al., 2013). Experimental evidence has also shown the *cstA* gene is upregulated during carbon starvation (Dubey et al., 2003) and allows for increased cellular growth by importing peptides as sources of C and N (Vastermark et al., 2014). Furthermore, it is possible that *N. moscoviensis* was genetically capable of responding to nutrient fluctuations in the no-mussel environment due to the glutathione-regulated potassium efflux gene (*kefC*) and methylglyoxal metabolism genetic markers. Evidence suggests that glutathione activates KefC potassium channels to protect against the toxic effects of methylglyoxal, and often occurs in response to limited phosphate or excessive carbon concentrations (Ferguson et al., 1998; Booth, 2005). It is possible that *N. moscoviensis* were C-stressed and nutrient-stressed in sediments without mussels, therefore the environment selected for the NOB *Nitrospira* possessing CstA and KefC proteins. Previous studies have shown that mussel biodeposits contain fairly consistent carbon and nutrient ratios (van Broekhoven et al., 2015) while others indicate biodeposit decomposition varies seasonally (Jansen et al., 2012), so future research should address if nutrient composition of mussel habitat sediments correspond to real-time metabolic activity of *N. moscoviensis*.

Furthermore, it is possible that mussel habitat sediments housed an enhanced niche for *N. moscoviensis* by containing a diverse source of carbon rather than nitrogen compounds. Contrary to our initial hypothesis, formate hydrogenase enzymes were a highly abundant N-cycling SEED category, while the gene families *nirK*, *ure* and *urt*, represented the lowest N-cycling genetic potential. These results suggest that *N. moscoviensis* had greater genetic potential to degrade to formate than from reciprocal feeding of mussel-derived urea. Taken together, these results signify *N. moscoviensis* in the mussel habitat had greatest potential for carbon degradation, ${\text{NO}}_{2}^{-}$ oxidation, and likely thrived from fermentation products commonly found in sediments at the interface of oxic and anoxic conditions.

### Comammox *Nitrospira* Marked by Potential for Multidrug Efflux Pumps and Diverse Metabolism

The comammox genome was marked with multidrug resistance efflux pumps (CmeB/AcrB), and metal resistance (Zn and Cu) in the mussel habitat, and suggests there may have been a selective pressure to use defense mechanisms. These multidrug resistance genes could be a response to one or more substrates and would enable resistance toward numerous antimicrobial substrates including antibiotics and heavy metals (Lin et al., 2002; Anes et al., 2015). Furthermore, our detected genetic markers for increased Phd-Doc toxin-antitoxin genes, are attributed with biochemical processes including antibiotic resistance (Liu et al., 2008). One explanation for these findings may be related to mussels hosting antibiotic resistant symbionts (Cooke, 1976; Liu et al., 2016), multidrug-resistant pathogens, (da Silveira et al., 2016), and enhanced horizontal gene transfer within their gut microbial community (Grevskott et al., 2017). Previous research has also shown that mussels bioaccumulate various metals (Naimo, 1995; Rzymski et al., 2014), including elements found in this genetic marker study, copper and zinc (Jorge et al., 2018). It is possible that horizontal gene transfer of multidrug resistance was facilitated by symbionts in mussel biodeposits, or from the decay of mussel tissue after death. However, this study was not designed to pinpoint antimicrobial stressors in the mussel habitat, so we cannot definitively say that commamox *Nitrospira* obtained these features as a direct result of their niche.

The genetic marker analysis also revealed that phosphorus metabolism was a distinctive feature of comammox *Nitrospira* in the mussel habitat. It is possible that phosphorus metabolic potential was influenced by mussel excretion products regenerating phosphate within the sediment, but would ultimately depend on the nutrient content and type of suspended food consumed by mussels (Jansen et al., 2012). Regeneration of phosphate in mussel habitat sediments would give comammox *Nitrospira* a selective advantage over AOA and AOB which do not encode an alkaline phosphatase (Palomo et al., 2018). Another genetic marker potentially explaining the success of comammox *Nitrospira* was the NiFe hydrogenase maturation protein (HypF) found in the mussel habitat. Recent studies have reported that comammox have the potential to oxidize H_2_ and use sulfur as an electron acceptor in anaerobic conditions (Camejo et al., 2017), and this further emphasizes the importance of metabolic versatility in the UMR mussel habitat.

### NOB and Comammox *Nitrospira* Coexisted in a Mussel Habitat Niche

Our results from the mussel habitat showed that “*Candidatus* Nitrospira inopinata” had the largest genomic potentials for ammonia oxidation, urea decomposition, and ${\text{NO}}_{2}^{-}$ redox reactions. In comparison to *N. moscoviensis*, “*Candidatus* Nitrospira inopinata” was genetically equipped to obtain electrons from multiple N compounds. Furthermore, since the comammox genome contained large abundances of N-cycling genes compared to *N. moscoviensis*, this suggests that these two organisms were likely successful in the mussel habitat by utilizing different metabolic functions. In the mussel habitat, “*Candidatus* Nitrospira inopinata” had an order of magnitude greater capacity for urea decomposition than *Nitrospira moscoviensis*, with the most evident differences shown for urease proteins and urea transporters. It is likely that the comammox *Nitrospira* genome had greater metabolic potentials for urea decomposition since it contains the whole gene set encoding a urea ABC transporter (*urtABCDE*). This high affinity urea transporter is shared among most *Nitrospira* species (Ushiki et al., 2017) and likely enables an adaptation to environments with variable and micromolar concentrations of urea. In comparison, *N. moscoviensis* only has the potential to encode UrtA, and not accessory proteins UreD, UreE, UreF, and UreG (Koch et al., 2015).

Not only do these results suggest that *Ca.* N. inopinata had a greater genetic potential to degrade urea, but also may have outcompeted the potential for reciprocal feeding between *N. moscoviensis* and other ammonia oxidizers. Another explanation for this finding could be that *Ca.* N. inopinata has over an order of magnitude greater affinity for NH_3_ than canonical AOB and is well suited for low NH_3_ and variable environments (Kits et al., 2017*)*. The importance of scavenging urea and NH_3_ is emphasized by the fact that “*Candidatus* Nitrospira inopinata” cannot survive on ${\text{NO}}_{2}^{-}$ alone, since the organism lacks the ability to utilize ${\text{NO}}_{2}^{-}$ as a source of N (Daims et al., 2015).

Although comammox *Nitrospira* would have the advantage over *N. moscovinesis* by scavenging NH_3_ and urea, both organisms could occupy the same niche due to their unique metabolic flexibilities. NOB *Nitrospira* thrive in oxic-anoxic niches where formate is produced by fermentation, and would not be outcompeted by comammox *Nitrospira* clade A, which lack a formate dehydrogenase enzyme (Hu and He, 2017). Furthermore, *N. moscovinesis* can simultaneously produce and consume ${\text{NO}}_{2}^{-}$, by coupling formate oxidation to ${\text{NO}}_{3}^{-}$ reduction while aerobically oxidizing ${\text{NO}}_{2}^{-}$ (Koch et al., 2015). This may explain our findings that *Nitrospira moscoviensis* had high genomic potentials for ${\text{NO}}_{2}^{-}$ oxidation (Nxr), ${\text{NO}}_{3}^{-}$reduction (NapG), and formate hydrogenation (Hyf, Fds).

Surprisingly, we detected an order of magnitude greater *nirK* compared to *nxr* in “*Candidatus* Nitrospira inopinata” and signifies that NO could be a major product of nitrification in this mussel habitat niche. Although previous studies have not documented gaseous NO_x_ production from nirK belonging to *Nitrospira*, comammox organisms do have the genetic capability for ${\text{NO}}_{3}^{-}$ reduction to ${\text{NO}}_{2}^{-}$ and NO (Camejo et al., 2017). Furthermore, *N. moscoviensis* showed genomic potential to respond to and degrade NO and N_2_O, potentially removing the major gaseous products of nitrification.

Our findings agree with other comparative genomic studies showing that *Nitrospira* species are functionally diverse and provides new insights on the niche separation between comammox and NOB (Hu and He, 2017). Although many genomic features differed between *N. moscoviensis* and *Ca*. N. inopinata in this study, both had greater genetic potentials for type IV pili in the mussel habitat. This feature would enhance the ability of these organisms to respond to environmental changes and form protective flocs and biofilms (Hospenthal et al., 2017). Aggregation of these two species is feasible and consistent with their ecophysiological versatility (Kits et al., 2017). Ultimately, our results indicated that NOB- and comammox-*Nitrospira* were genetically capable of coexisting in the mussel habitat through niche differentiation features, but potentially also synergistically coupled their metabolic features.

## Conclusion

This research used genomic markers to show that *Nitrospira moscoviensis* and “*Candidatus* Nitrospira inopinata” predominated an N-cycling oxic-anoxic niche in sediment of a mussel habitat. Our research showed that formate oxidation coupled to ${\text{NO}}_{3}^{-}$ reduction by NOB *Nitrospira* may have enabled co-existence with comammox *Nitrospira* in this sediment niche. The mussel habitat harbored comammox *Nitrospira* with enhanced RND efflux transporters and metal resistance, phosphorus metabolism, and showed evidence of hydrogen oxidation, while decreasing the genomic potential for potassium homeostasis and oxidative stress. For *N. moscoviensis*, the mussel habitat contained greater abundances of translational control genes and heme utilization while the no-mussel treatment showed genomic evidence of carbon stress. Both NOB and comammox *Nitrospira* were marked by diverse metabolism in the mussel habitat and may have contributed toward the increased abundance of both organisms. More research is needed to determine the biogeochemical signatures of the mussel habitat that may be responsible for these various genetic markers. Ultimately, this study provided metagenomic evidence showing that niche partitioning and mixotrophic metabolism allowed NOB and commamox *Nitrospira* to coexist in mussel habitat sediment.

## Author Contributions

EB and CJ contributed conception and design of the study. EB performed the statistical analysis and bioinformatics. EB and CJ wrote sections of the manuscript. All the authors contributed to manuscript revision, read and approved the submitted version.

## Conflict of Interest Statement

The authors declare that the research was conducted in the absence of any commercial or financial relationships that could be construed as a potential conflict of interest.

## References

[B1] Andrews S. (2010). *FastQC: A Quality Control tool for High Throughput Sequence Data.* Available at: http://www.bioinformatics.babraham.ac.uk/projects/fastqc

[B2] Anes J. McCusker M. P. Fanning S. Martins M. (2015). The ins and outs of RND efflux pumps in *Escherichia coli*. *Front. Microbiol.* 6:587 10.3389/fmicb.2015.00587 PMC4462101 26113845

[B3] Atkinson C. L. Kelly J. Vaughn C. C. (2014). Tracing consumer-derived nitrogen in riverine food webs. *Ecosystems* 17 485–496. 10.1007/s10021-013-9736-2

[B4] Atkinson C. L. Vaughn C. C. (2015). Biogeochemical hotspots: temporal and spatial scaling of the impact of freshwater mussels on ecosystem function. *Freshw. Biol.* 60 563–574. 10.1111/fwb.12498

[B5] Atkinson C. L. Vaughn C. C. Forshay J. K. Cooper J. T. (2013). Aggregated filter-feeding consumers alter nutrient limitation: consequences for ecosystem and community dynamics. *Ecology* 94 1359–1369. 10.1890/12-1531.1 23923499

[B6] Black E. M. Chimenti S. M. Just C. L. (2017). Effect of freshwater mussels on the vertical distribution of anaerobic ammonia oxidizers and other nitrogen-transforming microorganisms in upper Mississippi river sediment. *PeerJ* 5:e3536. 10.7717/peerj.3536 28717594 PMC5510576

[B7] Booth I. R. (2005). Glycerol and methylglyoxal metabolism. *EcoSal Plus.* 10.1128/ecosalplus.3.4.3 26443506

[B8] Bott C. B. Love N. G. (2004). Implicating the Glutathione-Gated Potassium Efflux System as a Cause of Electrophile-Induced Activated Sludge Deflocculation. *Appl. Environ. Microbiol.* 70 5569–5578. 10.1128/AEM.70.9.5569-5578.2004 15345445 PMC520847

[B9] Bril J. S. Durst J. J. Hurley B. M. Just C. L. Newton T. J. (2014). Sensor data as a measure of native freshwater mussel impact on nitrate formation and food digestion in continuous-flow mesocosms. *Freshw. Sci.* 33 417–424. 10.1086/675448

[B10] Bril J. S. Langenfeld K. Just C. L. Spak N. S. Newton T. J. (2017). Simulated mussel mortality thresholds as a function of mussel biomass and nutrient loading. *PeerJ* 5:e2838. 10.7717/peerj.2838 28070462 PMC5217613

[B11] Buchfink B. Xie C. Huson H. D. (2015). Fast and sensitive protein alignment using DIAMOND. *Nat. Methods* 12 59–60. 10.1038/nmeth.3176 25402007

[B12] Camejo P. Y. Santo J. Domingo D. McMahon K. D. Noguera R. (2017). Genome-enabled insights into the ecophysiology of the comammox bacterium *Candidatus* Nitrospira nitrosa. *mSystems* 2 e00059-17. 10.1128/mSystems.00059-17 28905001 PMC5596200

[B13] Cardenas J. P. Quatrini R. Holmes D. S. (2016). Aerobic lineage of the oxidative stress response protein rubrerythrin emerged in an ancient microaerobic, (hyper)thermophilic environment. *Front. Microbiol.* 7:1822. 10.3389/fmicb.2016.01822 27917155 PMC5114695

[B14] Carver T. Thomson N. Bleasby A. Berriman M. Parkhill J. (2009). DNAPlotter: circular and linear interactive genome visualization. *Bioinformatics* 25 119–120. 10.1093/bioinformatics/btn578 18990721 PMC2612626

[B15] Cooke M. D. (1976). Antibiotic Resistance Among Coliform and Fecal Coliform Bacteria Isolated from the Freshwater Mussel Hydridella menziesii. *Antimicrob. Agents Chemother.* 9 885–888. 10.1128/AAC.9.6.885 779633 PMC429644

[B16] da Silveira C. S. de Sousa O. V. Evangelista-Barreto N. S. (2016). Propagation of Antimicrobial Resistant *Salmonella* spp. *in* bivalve mollusks from estuary areas of bahia, brazil. *Rev. Caatinga* 29 450–457. 10.1590/1983-21252016v29n222rc

[B17] Daebeler A. Bodelier P. L. E. Yan Z. Hefting M. M. Jia Z. Laanbroek H. J. (2014). Interactions between Thaumarchaea, *Nitrospira* and methanotrophs modulate autotrophic nitrification in volcanic grassland soil. *ISME J.* 8:2397. 10.1038/ismej.2014.81 24858784 PMC4260704

[B18] Daims H. Lebedeva E. V. Pjevac P. Han P. Herbold C. Albertsen M. (2015). Complete nitrification by *Nitrospira* bacteria. *Nature* 528 504–509. 10.1038/nature16461 26610024 PMC5152751

[B19] Danovaro R. Gambi C. Luna G. M. Mirto S. (2004). “Sustainable impact of mussel farming in the Adriatic Sea (Mediterranean Sea): evidence from biochemical, microbial and meiofaunal indicators”. *Mar*. *Pollut. Bull.* 49 325–333. 10.1016/j.marpolbul.2004.02.038 15341827

[B20] Donner S. D. Kucharik C. J. (2008). “Corn-based ethanol production compromises goal of reducing nitrogen export by the Mississippi River”.*Proc*. *Natl. Acad. Sci. U.S.A.* 105 4513–4518. 10.1073/pnas.0708300105 PMC2393748 18332435

[B21] Dubey A. K. Baker C. S. Suzuki K. Jones A. D. Pandit P. Romeo T. (2003). CsrA regulates translation of the *Escherichia coli* carbon starvation gene, cstA, by blocking ribosome access to the cstA transcript. *J. Bacteriol.* 185 4450–4460. 10.1128/JB.185.15.4450-4460.2003 12867454 PMC165747

[B22] Ferguson G. P. McLaggan D. Booth I. R. (1995). Potassium channel activation by glutathione-S-conjugates in *Escherichia coli*: protection against methylglyoxal is mediated by cytoplasmic acidification. *Mol. Microbiol.* 17 1025–1033. 10.1111/j.1365-2958.1995.mmi_17061025.x 8594323

[B23] Ferguson G. P. Totemeyer S. MacLean M. J. Booth I. R. (1998). “Methylglyoxal production in bacteria: suicide or survival?” *Arch. Microbiol.* 170 209–219. 10.1007/s002030050635 9732434

[B24] Gomez-Velez J. D. Harvey J. W. Cardenas M. B. Kiel B. (2015). Denitrification in the Mississippi River network controlled by flow through river bedforms. *Nat. Geosci.* 8:941 10.1038/ngeo2567

[B25] Grevskott D. H. Svanevik C. S. Sunde M. Wester A. L. Lunestad B. T. (2017). Marine bivalve mollusks as possible indicators of multidrug-resistant *Escherichia coli* and other species of the *Enterobacteriaceae* family. *Front. Microbiol.* 8:10. 10.3389/fmicb.2017.00024 28149295 PMC5241299

[B26] Hallin S. Jones C. M. Schloter M. Philippot L. (2009). Relationship between N-cycling communities and ecosystem functioning in a 50-year-old fertilization experiment. *ISME J.* 3 597–605. 10.1038/ismej.2008.128 19148144

[B27] Hershey J. W. B. Sonenberg N. Mathews M. B. (2012). Principles of translational control: an overview. *Cold Spring Harb. Perspect. Biol.* 4:a011528. 10.1101/cshperspect.a011528 23209153 PMC3504442

[B28] Hospenthal M. K. Costa D. T. R. Waksman G. (2017). A comprehensive guide to pilus biogenesis in Gram-negative bacteria. *Nat. Rev. Microbiol.* 15 365–379. 10.1038/nrmicro.2017.40 28496159

[B29] Hu H. W. He Z. J. (2017). Comammox—a newly discovered nitrification process in the terrestrial nitrogen cycle. *J. Soils Sediments* 17 2709–2717. 10.1007/s11368-017-1851-9

[B30] Huson D. H. Auch A. F. Qi J. Schuster S. C. (2007). MEGAN analysis of metagenomic data. *Genome Res.* 17 377–386. 10.1101/gr.5969107 17255551 PMC1800929

[B31] Huson D. H. Beier S. Flade I. Gorska A. El Hadidi M. Mitra S. (2016). MEGAN community edition - interactive exploration and analysis of large-scale microbiome sequencing data. *PLoS Comput. Biol.* 12:e1004957. 10.1371/journal.pcbi.1004957 27327495 PMC4915700

[B32] Irzik K. Pfrötzschner J. Goss T. Ahnert F. Haupt M. Greie C. J. (2011). The KdpC subunit of the *Escherichia coli* K+-transporting KdpB P-type ATPase acts as a catalytic chaperone. *FEBS J.* 278 3041–3053. 10.1111/j.1742-4658.2011.08224.x 21711450

[B33] Jansen H. M. Verdegem M. C. J. Strand Ø Smaal A. C. (2012). Seasonal variation in mineralization rates (C-N-P-Si) of mussel Mytilus edulis biodeposits. *Mar. Biol.* 159 1567–1580. 10.1007/s00227-012-1944-3 24391273 PMC3873025

[B34] Jorge M. B. Bianchini A. Wood M. C. Gillis P. L. (2018). Copper uptake, patterns of bioaccumulation, and effects in glochida (larvae) of the freshwater mussel (*Lampsilis cardium*). *Environ. Toxicol. Chem.* 37 1092–1103. 10.1002/etc.4041 29139577

[B35] Kits K. D. Sedlacek C. J. Lebedeva E. V. Han P. Bulaev A. Pjevac P. (2017). Kinetic analysis of a complete nitrifier reveals an oligotrophic lifestyle. *Nature* 549 269–272. 10.1038/nature23679 28847001 PMC5600814

[B36] Koch H. Galushko A. Albertsen M. Schintlmeister A. Gruber C. Dorninger S. (2014). Growth of nitrite-oxidizing bacteria by aerobic hydrogen oxidation. *Science* 345 1052–1054. 10.1126/science.1256985 25170152

[B37] Koch H. Lücker S. Albertsen M. Kitzinger K. Herbold C. Spieck E. (2015). Expanded metabolic versatility of ubiquitous nitrite-oxidizing bacteria from the genus *Nitrospira*. *Proc. Natl. Acad. Sci. U.S.A.* 112 11371–11376. 10.1073/pnas.1506533112 26305944 PMC4568715

[B38] Kreiling R. M. Richardson W. B. Cavanaugh J. C. Bartsch L. A. (2011). Summer nitrate uptake and denitrification in an upper Mississippi River backwater lake: the role of rooted aquatic vegetation. *Biogeochemistry* 104 309–324. 10.1007/s10533-010-9503-9

[B39] Kuypers M. M. M. Marchant K. H. Kartal B. (2018). The microbial nitrogen-cycling network. *Nat. Rev. Microbiol.* 16 263–276. 10.1038/nrmicro.2018.9 29398704

[B40] Lerch R. N. Kitchen N. R. Baffaut C. Vories E. D. (2015). Long-term agroecosystem research in the central mississippi river basin: goodwater creek experimental watershed and regional nutrient water quality data. *J. Environ. Qual.* 44 37–43. 10.2134/jeq2013.12.0518 25602319

[B41] Li H. Durbin R. (2009). Fast and accurate short read alignment with Burrows–Wheeler transform. *Bioinformatics* 25 1754–1760. 10.1093/bioinformatics/btp324 19451168 PMC2705234

[B42] Li H. Handsaker B. Wysoker A. Fennell T. Ruan J. Homer N. G. (2009). The sequence alignment/map format and SAMtools. *Bioinformatics* 25 2078–2079. 10.1093/bioinformatics/btp352 19505943 PMC2723002

[B43] Lin J. Michel L. O. Zhang Q. (2002). CmeABC Functions as a Multidrug Efflux System in Campylobacter jejuni. *Antimicrob. Agents Chemother.* 46 2124–2131. 10.1128/AAC.46.7.2124-2131.2002 12069964 PMC127319

[B44] Liu J. Jung J. H. Liu Y. H. (2016). Antimicrobial compounds from marine invertebrates-derived microorganisms. *Curr. Med. Chem* 23 2892–2905. 10.2174/0929867323666160525113837 27222267

[B45] Liu M. Zhang Y. Inouye M. N. Woychik A. (2008). Bacterial addiction module toxin Doc inhibits translation elongation through its association with the 30S ribosomal subunit. *Proc. Natl. Acad. Sci. U.S.A.* 105 5885–5890. 10.1073/pnas.0711949105 18398006 PMC2311363

[B46] Lücker S. Wagner M. Maixner F. Pelletier E. Koch H. Vacherie B. T. (2010). “A *Nitrospira* metagenome illuminates the physiology and evolution of globally important nitrite-oxidizing bacteria”. *Proc*. *Natl. Acad. Sci. U.S.A.* 107 13479–13484. 10.1073/pnas.1003860107 20624973 PMC2922143

[B47] Matantseva O. V. Skarlato S. O. (2013). Mixotrophy in microorganisms: ecological and cytophysiological aspects. *J. Evol. Biochem. Physiol.* 49 377–388. 10.1134/S0022093013040014 24459857

[B48] McGill B. J. Etienne R. S. Gray J. S. Alonso D. Anderson M. J. Benecha H. K. (2007). Species abundance distributions: moving beyond single prediction theories to integration within an ecological framework. *Ecol. Lett.* 10 995–1015. 10.1111/j.1461-0248.2007.01094.x 17845298

[B49] Mirto S. La rosa T. Danovaro R. Mazzola A. (2000). Microbial and Meiofaunal Response to Intensive Mussel-Farm Biodeposition in Coastal Sediments of the Western Mediterranean. *Mar. Pollut. Bull.* 40 244–252. 10.1016/S0025-326X(99)00209-X

[B50] Morales Y. Weber L. J. Mynett A. E. Newton T. J. (2006). Effects of substrate and hydrodynamic conditions on the formation of mussel beds in a large river. *J. North Am. Benthol. Soc.* 25 664–676. 10.1899/0887-3593(2006)25[664:EOSAHC]2.0.CO;2

[B51] Mulholland P. J. Helton A. M. Poole G. C. Hall R. O. Hamilton S. K. Peterson B. J. (2008). Stream denitrification across biomes and its response to anthropogenic nitrate loading. *Nature* 452:202. 10.1038/nature06686 18337819

[B52] Naimo T. J. (1995). A review of the effects of heavy metals on freshwater mussels. *Ecotoxicology* 4 341–362. 10.1007/BF00118870 24197828

[B53] Newton T. J. Zigler S. J. Rogala J. T. Gray B. R. Davis M. (2011). Population assessment and potential functional roles of native mussels in the Upper Mississippi River. *Aquat. Cons.* 21 122–131. 10.1002/aqc.1170

[B54] Okonechnikov K. Golosova O. Fursov M. (2012). Unipro UGENE: a unified bioinformatics toolkit. *Bioinformatics* 28 1166–1167. 10.1093/bioinformatics/bts091 22368248

[B55] O’Leary N. A. Wright M. W. Brister J. R. Ciufo S. Haddad D. McVeigh R. (2016). Reference sequence (RefSeq) database at NCBI: current status, taxonomic expansion, and functional annotation. *Nucleic Acids Res.* 44 D733–D745. 10.1093/nar/gkv1189 26553804 PMC4702849

[B56] Overbeek R. Begley T. Butler R. M. Choudhuri J. V. Chuang Y. H. Cohoon M. (2005). The Subsystems Approach to Genome Annotation and its Use in the Project to Annotate 1000 Genomes. *Nucleic Acids Res.* 33 5691–5702. 10.1093/nar/gki866 16214803 PMC1251668

[B57] Palatinszky M. Herbold C. Jehmlich N. Pogoda M. Han P. von Bergen M. (2015). Cyanate as energy source for nitrifiers. *Nature* 524 105–108. 10.1038/nature14856 26222031 PMC4539577

[B58] Palomo A. Pedersen A. G. Fowler S. J. Dechesne A. Sicheritz Pontén T. Smets B. F. (2018). Comparative genomics sheds differentiation light on niche comammox the evolutionary history of *Nitrospira*. *ISME J.* 12 1779–1793. 10.1038/s41396-018-0083-3 29515170 PMC6018701

[B59] Pfister C. A. Gilbert J. A. Gibbons S. M. (2014). The role of macrobiota in structuring microbial communities along rocky shores. *PeerJ* 2:e631. 10.7717/peerj.631 25337459 PMC4203024

[B60] Rasmussen J. J. Vegge C. S. Frokiaer H. Howlett R. M. Krogfelt K. A. Kelly D. J. (2013). Campylobacter jejuni carbon starvation protein A (CstA) is involved in peptide utilization, motility and agglutination, and has a role in stimulation of dendritic cells. *J. Med. Microbiol.* 62 1135–1143. 10.1099/jmm.0.059345-0 23682166

[B61] Rzymski P. Niedzielski P. Klimaszyk P. Poniedziałek B. (2014). Bioaccumulation of selected metals in bivalves (Unionidae) and Phragmites australis inhabiting a municipal water reservoir. *Environ. Monit. Assess* 186 3199–3212. 10.1007/s10661-013-3610-8 24407963 PMC3969812

[B62] Segata N. Izard J. Waldron L. Gevers D. Miropolsky L. Garrett W. S. (2011). Metagenomic biomarker discovery and explanation. *Genome Biol.* 12:R60. 10.1186/gb-2011-12-6-r60 21702898 PMC3218848

[B63] Shange R. S. Ankumah R. O. Ibekwe A. M. Zabawa R. Dowd S. E. (2012). Distinct soil bacterial communities revealed under a diversely managed agroecosystem. *PLoS One* 7:11. 10.1371/journal.pone.0040338 22844402 PMC3402512

[B64] Spieck E. Ehrich S. Aamand J. Bock E. (1998). Isolation and immunocytochemical location of the nitrite-oxidizing system in Nitrospira moscoviensis. *Arch. Microbiol.* 169 225–230. 10.1007/s002030050565 9477257

[B65] Strauss E. A. Richardson W. B. Cavanaugh J. C. Bartsch L. A. Kreiling R. M. Standorf A. J. (2006). Variability and regulation of denitrification in an Upper Mississippi River backwater. *J. North Am. Benthol. Soc.* 25 596–606. 10.1899/0887-3593(2006)25[596:VARODI]2.0.CO;2

[B66] Teplyakov A. Obmolova G. Chu S. Y. Toedt J. Eisenstein E. Howard A. J. (2003). Crystal Structure of the YchF Protein Reveals Binding Sites for GTP and Nucleic Acid. *J. Bacteriol.* 185 4031–4037. 10.1128/JB.185.14.4031-4037.2003 12837776 PMC164861

[B67] Tötemeyer S. Booth N. A. Nichols W. W. Dunbar B. Booth I. R. (1998). From famine to feast: the role of methylglyoxal production in *Escherichia coli*. *Mol. Microbiol.* 27 553–562. 10.1046/j.1365-2958.1998.00700.x 9489667

[B68] USACE (1981). *Survey of Freshwater Mussels (Pelecypoda: Unionidae) at Selected Sites in Pools 11 Through 24 of the Mississippi River.* Illinois, IL: EA Report 9031 Rock Island District.

[B69] USACE (1984). *Resources Inventory for the Upper Mississippi River Rock Island District.* Illinois, IL: US Army Corps of Engineers.

[B70] Ushiki N. Fujitani H. Shimada Y. Morohoshi T. Sekiguchi Y. Tsuneda S. (2017). Genomic analysis of two phylogenetically distinct *Nitrospira* species reveals their genomic plasticity and functional diversity. *Front. Microbiol.* 8:2637. 10.3389/fmicb.2017.02637 29375506 PMC5767232

[B71] van Broekhoven W. Jansen H. Verdegem M. Struyf E. Troost K. Lindeboom H. (2015). Nutrient regeneration from feces and pseudofeces of mussel *Mytilus edulis* spat. *Mar. Ecol. Prog. Ser.* 534 107–120. 10.3354/meps11402

[B72] van Kessel M. A. Speth D. R. Albertsen M. Nielsen P. H. Op den Camp J. M. Kartal B. (2015). Complete nitrification by a single microorganism. *Nature* 528 555. 10.1038/nature16459 26610025 PMC4878690

[B73] Vastermark A. Wollwage S. Houle M. E. Rio R. Saier M. H. (2014). Expansion of the APC superfamily of secondary carriers. *Prot. Struct. Funct. Bioinform.* 82 2797–2811. 10.1002/prot.24643 25043943 PMC4177346

[B74] Vaughn C. C. Hakenkamp C. C. (2001). The functional role of burrowing bivalves in freshwater ecosystems. *Freshw. Biol.* 46 1431–1446. 10.1046/j.1365-2427.2001.00771.x

[B75] Young N. Andrew M. Weber L. (2005). “Hydrodynamic Investigation of Upper Mississippi River Freshwater Mussel Habitats,” in *Proceedings of the American Society of Civil Engineers World Water and Environmental Resources Congress*, Anchorage, AL 10.1061/40792(173)585

[B76] Zhang Y. Lin J. Gao Y. (2012). In silico identification of a multi-functional regulatory protein involved in Holliday junction resolution in bacteria. *BMC Syst. Biol.* 6:S20. 10.1186/1752-0509-6-S1-S20 23046553 PMC3403352

[B77] Zhou X. L. Chen Y. Fang Z. P. Ruan Z. R. Wang Y. Liu R. J. (2016). Translational quality control by bacterial threonyl-tRNA synthetases. *J. Biol. Chem.* 291 21208–21221. 10.1074/jbc.M116.740472 27542414 PMC5076528

