# Supplementary material for: The Genomic Potentials of NOB and Comammox Nitrospira in River Sediment Are Impacted by Native Freshwater Mussels
Source: Front Microbiol. 2018 Sep 4;9:2061. doi: 10.3389/fmicb.2018.02061 (PMC6131200; doi:10.3389/fmicb.2018.02061)
Supplement: Supplementary file 1 [file Table_1.docx]

Supplementary Material

The genomic potentials of NOB and comammox *Nitrospira* in river sediment are impacted by native freshwater mussels

Ellen M. Black, Craig L. Just*

***Correspondence:** Craig-Just@uiowa.edu

# Supplementary Tables

Table S1: Most abundant protein functions for *Nitrospira moscovinesis* metagenomic reads, listed as a percentage of total reads with a SEED classification.

| Top 25% SEED subsystems | | | |
| --- | --- | --- | --- |
| **No Mussels** | | **Mussel Habitat** | |
| Peptidoglycan Biosynthesis (mur/ddl genes) | 3.00% | Peptidoglycan Biosynthesis  (mur/ddl genes) | 3.12% |
| Respiratory Complex I | 2.65% | Respiratory Complex I | 3.09% |
| Entner-Doudoroff Pathway | 2.49% | Bacterial Cytoskeleton | 2.22% |
| Bacterial Cytoskeleton | 2.23% | Multidrug Resistance Efflux Pumps | 2.18% |
| DNA-replication | 2.17% | Entner-Doudoroff Pathway | 1.91% |
| Multidrug Resistance Efflux Pumps | 2.15% | DNA-replication | 1.84% |
| DNA repair, bacterial | 1.74% | DNA repair, bacterial | 1.76% |
| 5-FCL-like protein | 1.60% | Folate Biosynthesis | 1.72% |
| EC 6.1.1.- Ligases forming aminoacyl-tRNA and related compounds | 1.60% | Cysteine Biosynthesis | 1.70% |
| Biotin biosynthesis | 1.55% | Proteasome bacterial | 1.68% |
| Bacterial Chemotaxis | 1.40% | EC 6.1.1.- Ligases forming aminoacyl-tRNA and related compounds | 1.55% |
| Glutathione-regulated potassium-efflux system and associated functions | 1.38% | YebC | 1.39% |
| Bacterial motility:Gliding | 1.33% | alpha carboxysome | 1.37% |
|  |  |  |  |
| **Top 25% SEED enzymes** | | | |
| **No Mussels** | | **Mussel Habitat** | |
| Acriflavin resistance protein | 1.85% | Acriflavin resistance protein | 1.92% |
| Gluconolactonase (EC 3.1.1.17) | 1.18% | ATP-dependent protease  (EC 3.4.21.53) Type I | 1.67% |
| Carbon starvation protein A | 1.14% | Type II secretory pathway, ATPase PulE/Tfp pilus assembly pathway, ATPase PilB | 1.02% |
| Response regulator of zinc sigma-54-dependent two-component system | 1.08% | Threonyl-tRNA synthetase (EC 6.1.1.3) | 0.99% |
| Type II secretory pathway, ATPase PulE/Tfp pilus assembly pathway, ATPase PilB | 1.00% | Carbon starvation protein A | 0.92% |
| Long-chain-fatty-acid--CoA ligase  (EC 6.2.1.3) | 0.98% | NADH-ubiquinone oxidoreductase chain G (EC 1.6.5.3) | 0.92% |
| 1-deoxy-D-xylulose 5-phosphate synthase (EC 2.2.1.7) | 0.93% | Thymidylate synthase thyX  (EC 2.1.1.-) | 0.87% |
| Glucose-6-phosphate 1-dehydrogenase  (EC 1.1.1.49) | 0.91% | Gluconolactonase (EC 3.1.1.17) | 0.87% |
| ATP-dependent protease La (EC 3.4.21.53) Type I | 0.83% | Long-chain-fatty-acid--CoA ligase  (EC 6.2.1.3) | 0.85% |
| NADH-ubiquinone oxidoreductase chain G (EC 1.6.5.3) | 0.80% | Response regulator of zinc sigma-54-dependent two-component system | 0.81% |
| Phosphate regulon sensor protein PhoR (SphS) (EC 2.7.13.3) | 0.78% | NADH-ubiquinone oxidoreductase chain M (EC 1.6.5.3) | 0.79% |
| Multimodular transpeptidase-transglycosylase (EC 2.4.1.129)  (EC 3.4.-.-) | 0.74% | Phosphate regulon sensor protein PhoR (SphS) (EC 2.7.13.3) | 0.79% |
| Adenylylsulfate kinase (EC 2.7.1.25) | 0.70% | Glucose-6-phosphate 1-dehydrogenase (EC 1.1.1.49) | 0.77% |
| Signal transduction histidine kinase CheA  (EC 2.7.3.-) | 0.69% | ATP-dependent DNA helicase UvrD/PcrA, proteobacterial paralog | 0.76% |
| Hypothetical transmembrane protein coupled to NADH-ubiquinone oxidoreductase homolog (chain L) | 0.67% | Hypothetical transmembrane protein coupled to NADH-ubiquinone oxidoreductase homolog (chain L) | 0.70% |
| diguanylate cyclase/phosphodiesterase (GGDEF & EAL domains) with PAS/PAC sensor(s) | 0.66% | Adenylylsulfate kinase (EC 2.7.1.25) | 0.70% |
| tRNA pseudouridine 13 synthase (EC 4.2.1.-) | 0.66% | Single-stranded-DNA-specific exonuclease RecJ (EC 3.1.-.-) | 0.69% |
| Soluble pyridine nucleotide transhydrogenase (EC 1.6.1.1) | 0.65% | Deoxyhypusine synthase (EC 2.5.1.46) | 0.67% |
| DNA mismatch repair protein MutS | 0.64% | Peptidyl-prolyl cis-trans isomerase  (EC 5.2.1.8) | 0.66% |
| Asparagine synthetase [glutamine-hydrolyzing] (EC 6.3.5.4) | 0.63% | Soluble pyridine nucleotide transhydrogenase (EC 1.6.1.1) | 0.65% |
| Deoxyhypusine synthase (EC 2.5.1.46) | 0.62% | Sulfate adenylyltransferase subunit 2 (EC 2.7.7.4) | 0.65% |
| NADH-ubiquinone oxidoreductase chain M (EC 1.6.5.3) | 0.61% | D-alanine--D-alanine ligase  (EC 6.3.2.4) | 0.64% |
| Acetylornithine aminotransferase  (EC 2.6.1.11) | 0.61% | Multimodular transpeptidase-transglycosylase (EC 2.4.1.129)  (EC 3.4.-.-) | 0.63% |
| Peptidyl-prolyl cis-trans isomerase  (EC 5.2.1.8) | 0.59% | DNA mismatch repair protein MutS | 0.61% |
| DNA recombination protein RmuC | 0.59% | Signal transduction histidine kinase CheA (EC 2.7.3.-) | 0.59% |
| Chaperone protein DnaJ | 0.59% | TldD family protein, Actinobacterial subgroup | 0.58% |
| Chromosomal replication initiator protein DnaA | 0.58% | Thymidylate kinase (EC 2.7.4.9) | 0.58% |
| Thymidylate synthase thyX (EC 2.1.1.-) | 0.57% | diguanylate cyclase/phosphodiesterase (GGDEF & EAL domains) with PAS/PAC sensor(s) | 0.57% |
| tRNA nucleotidyltransferase (EC 2.7.7.21) (EC 2.7.7.25) | 0.55% | DNA recombination protein RmuC | 0.55% |
| 3-oxoacyl-[acyl-carrier protein] reductase  (EC 1.1.1.100) | 0.55% | Phenylalanyl-tRNA synthetase alpha chain (EC 6.1.1.20) | 0.55% |
| Superoxide dismutase [Mn] (EC 1.15.1.1) | 0.54% | Acetylornithine aminotransferase  (EC 2.6.1.11) | 0.54% |
| TldD family protein, Actinobacterial subgroup | 0.52% | Cell division protein FtsZ (EC 3.4.24.-) | 0.54% |
| DNA polymerase III alpha subunit  (EC 2.7.7.7) | 0.52% |  | |
| Ribosomal protein S12p Asp88 (E. coli) methylthiotransferase | 0.51% |  |  |

Table S2: Protein functions with statistically significant LDA effect sizes for *Nitrospira moscoviensis*.

| **Treatment** | **SEED classification** | **LDA effect size** | **P-value** | **N. Moscoviensis gene ID** | **N. moscoviensis gene name** |
| --- | --- | --- | --- | --- | --- |
| Mussel Habitat | YebC-like protein (EC 6.1.1.3) | 3.49 | 0.043 | NITMOv2_0644; NITMOv2_0329 | YchF and thrS |
|  | hypothetical protein related to heme utilization (FIG039061) | 3.33 | 0.021 | - | - |
|  | ATP dependent DNA helicase (UvrD/PcrA) proteobacterial paralog | 3.28 | 0.043 | NITMOv2_1186 | PcrA |
|  | Histidinol dehydrogenase (EC 1.1.1.23) | 2.91 | 0.021 | NITMOv2_0825 | hisD |
|  | Ribulose phosphate-3-epimerase  (EC 5.1.3.1) | 2.87 | 0.021 | NITMOv2_0323 | cbbE |
|  | Ribonuclease H III (EC 3.1.26.4) | 2.85 | 0.043 | NITMOv2_0850 | rnhC |
|  | Type IV pilus biogenesis protein (PilQ) | 2.84 | 0.043 | NITMOv2_1246 | pilQ |
|  | Signal recognition particle subunit (Ffh) SRP54 (EC 3.A.5.1.1) | 2.76 | 0.043 | NITMOv2_3569 | ffh |
|  | 4-alpha-glucanotransferase amylomaltase (EC 2.4.1.25) | 2.74 | 0.043 | NITMOv2_1162 | malQ |
|  | GMP synthase (glutamine-hydrolyzing)  (EC 6.3.5.2) | 2.68 | 0.021 | NITMOv2_1344 | guaA |
|  | Alkaline phosphatase (EC 3.1.3.1) | 2.67 | 0.021 | NITMOv2_2620 | PhoP |
| No Mussel | Glutathione regulated potassium efflux system | 3.31 | 0.021 | NITMOv2_4520 | CPA2 protein family (i.e. KefB) |
|  | Phosphogluconolactonase | 3.16 | 0.043 | NITMOv2_0274 | pgl |
|  | Misc. protein function (COG2363) | 3.10 | 0.021 | - | integral membrane protein of unknown function DUF423 with Metl-like superfamily |
|  | Superoxide dismutase Fe-Mn (EC 1.15.1.1) | 3.10 | 0.043 | NITMOv2_2805 | sod |
|  | Asparagine synthetase (glutamine-hydrolyzing) (EC.6.3.5.4) | 3.06 | 0.021 | NITMOv2_3400 | asnB |
|  | Glucose methanol choline (GMC) oxidoreductase NAD binding site | 3.04 | 0.043 | NITMOv2_4007 | gmc |
|  | Pyruvate decarboxylase (EC 4.1.1.1) | 3.04 | 0.021 | NITMOv2_3169 | cfp |
|  | Carbon starvation protein A | 3.00 | 0.021 | NITMOv2_0147 | cstA |
|  | Beta hexosaminidase (EC 3.2.1.52) | 2.94 | 0.043 | NITMOv2_0180 | nagZ |
|  | Tryptophanase (EC 4.1.99.1) | 2.83 | 0.021 | NITMOv2_0067 | tnaA |
|  | Ribosomal large subunit pseudouridine synthase D (EC 4.2.1.70) | 2.76 | 0.043 | NITMOv2_0475 | truD |
|  | Endonuclease III (EC 4.2.99.18) | 2.72 | 0.021 | NITMOv2_4513 | nth |
|  | Xylulose-5-phosphate phosphoketolase  (EC 4.1.2.9) | 2.72 | 0.043 | NITMOv2_2536 | xfp |
|  | Flagellar biosynthesis protein (FlhB) | 2.72 | 0.043 | NITMOv2_2196 | flhB |
|  | Acetoacetate metabolism regulatory protein (AtoC) | 2.70 | 0.021 | NITMOv2_1164; NITMOv2_1908; NITMOv2_2568; NITMOv2_3235; NITMOv2_3750; NITMOv2_4352 | AtoC |

Table S3: Most abundant protein functions for *Candidatus* Nitrospira inopinata metagenomic reads, listed as a percentage of total reads with a SEED classification.

| **Top 25% SEED subsystems** | | | |
| --- | --- | --- | --- |
| **No Mussels** | | **Mussel Habitat** | |
| Restriction-Modification System | 5.56% | 5-FCL-like protein | 4.34% |
| Urea decomposition | 4.00% | FOL Commensurate regulon activation | 4.34% |
| 5-FCL-like protein | 3.92% | Biogenesis of c-type cytochromes | 4.27% |
| Ammonia monooxygenase | 3.65% | Ammonia monooxygenase | 3.79% |
| Biogenesis of c-type cytochromes | 3.48% | Urea decomposition | 3.52% |
| FOL Commensurate regulon activation | 3.26% | Restriction-Modification System | 3.16% |
| Glycogen metabolism | 2.56% | Glycogen metabolism | 2.83% |
| **Top 25% most abundant SEED enzymes** | | | |
| **No Mussels** | | **Mussel Habitat** | |
| RND efflux system, inner membrane transporter CmeB | 3.26% | RND efflux system, inner membrane transporter CmeB | 4.34% |
| Type I restriction-modification system, restriction subunit R (EC 3.1.21.3) | 3.10% | Ammonia monooxygenase C-subunit  (EC 1.14.13.25) | 2.29% |
| Ammonia monooxygenase C-subunit  (EC 1.14.13.25) | 2.51% | Alcohol dehydrogenase (EC 1.1.1.1) | 1.83% |
| Osmosensitive K+ channel histidine kinase KdpD (EC 2.7.3.-) | 1.80% | Type I restriction-modification system, restriction subunit R (EC 3.1.21.3) | 1.69% |
| Alcohol dehydrogenase (EC 1.1.1.1) | 1.68% | 1,4-alpha-glucan (glycogen) branching enzyme, GH-13-type (EC 2.4.1.18) | 1.51% |
| DNA-directed RNA polymerase beta' subunit (EC 2.7.7.6) | 1.38% | Cytochrome c heme lyase subunit CcmF | 1.45% |
| Cytochrome c heme lyase subunit CcmF | 1.27% | Osmosensitive K+ channel histidine kinase KdpD (EC 2.7.3.-) | 1.34% |
| 1,4-alpha-glucan (glycogen) branching enzyme, GH-13-type (EC 2.4.1.18) | 1.24% | Cobalt-zinc-cadmium resistance protein CzcA | 1.30% |
| Type III restriction-modification system methylation subunit (EC 2.1.1.72) | 1.15% | Cytochrome c551 peroxidase (EC 1.11.1.5) | 1.30% |
| Putative two-domain glycosyltransferase | 1.13% | Glycogen phosphorylase (EC 2.4.1.1) | 1.14% |
| Glycogen phosphorylase (EC 2.4.1.1) | 1.10% | Ferredoxin | 1.10% |
| Archaeal S-adenosylmethionine synthetase (EC 2.5.1.6) | 1.08% | Putative two-domain glycosyltransferase | 1.04% |
| Long-chain-fatty-acid Acetyl-CoA ligase (EC 6.2.1.3) | 1.06% | Exopolyphosphatase (EC 3.6.1.11) | 1.03% |
| Cobalt-zinc-cadmium resistance protein CzcA | 1.05% | Archaeal S-adenosylmethionine synthetase  (EC 2.5.1.6) | 1.01% |
| DNA recombination protein RmuC | 1.02% | [NiFe] hydrogenase metallocenter assembly protein HypF | 0.98% |
| Cytochrome c551 peroxidase  (EC 1.11.1.5) | 0.95% | Urea ABC transporter, permease protein UrtB | 0.93% |
| Urea ABC transporter, urea binding protein | 0.95% | Asparagine synthetase [glutamine-hydrolyzing] (EC 6.3.5.4) | 0.92% |

Table S4: Protein functions with statistically significant effect sizes for *Candidatus* Nitrospira inopinata.

| **Treatment** | **SEED classification** | **LDA effect size** | **P-value** | ***Ca.* N. inopinata ID** | ***Ca.* N. inopinata gene name** |
| --- | --- | --- | --- | --- | --- |
| Mussel Habitat | RND efflux system inner membrane transporter (CmeB) | 3.81 | 0.043 | NITINOP_0165; NITINOP_0322 | acrB |
|  | NiFe hydrogenase metallocenter assembly protein (HypF) | 3.21 | 0.043 | NITINOP_0583 | hypF |
|  | Thiamine-monophosphate kinase | 3.11 | 0.021 | [NITINOP_3242; NITINOP_3243](http://www.genome.jp/dbget-bin/www_bget?nio:NITINOP_3141) | thiE; thiG |
|  | Cytochrome c type biogenesis protein (CcmE) heme chaperone | 3.00 | 0.043 | [NITINOP_0071](http://www.genome.jp/dbget-bin/www_bget?nio:NITINOP_0071) | ccmE |
|  | Lipopolysaccharide heptosyltransferase I  (EC 2.4.1.-) | 2.89 | 0.043 | [NITINOP_3119](http://www.genome.jp/dbget-bin/www_bget?nio:NITINOP_3124) | putative ADP-heptose-LPS heptosyltransferase |
|  | Threonine synthase (EC 4.2.3.1) | 2.45 | 0.014 | [NITINOP_0769](http://www.genome.jp/dbget-bin/www_bget?nio:NITINOP_0769) | thrC |
|  | Phosphoribosylformylglycinamidine synthase (EC 6.3.5.3) | 2.37 | 0.014 | [NITINOP_1803](http://www.genome.jp/dbget-bin/www_bget?nio:NITINOP_1800) | purL |
|  | A/G specific adenine glycosylase (EC 3.2.2.-) | 2.36 | 0.047 | [NITINOP_0052](http://www.genome.jp/dbget-bin/www_bget?nio:NITINOP_2029) | mutS |
|  | HflK membrane protease (EC 3.4.-.-) | 2.35 | 0.047 | NITINOP_0656; NITINOP_2485 | ftsH |
|  | Carbamoyl phosphate synthase large chain  (EC 6.3.5.5) | 2.35 | 0.047 | [NITINOP_2878](http://www.genome.jp/dbget-bin/www_bget?nio:NITINOP_2878) | carB |
|  | Sensor protein of zinc sigma-54-dependent two component system | 2.33 | 0.047 | [NITINOP_0732](http://www.genome.jp/dbget-bin/www_bget?nio:NITINOP_0732) | kdpD |
|  | Periplasmic divalent cation tolerance protein (cutA) | 2.23 | 0.047 | [NITINOP_1511](http://www.genome.jp/dbget-bin/www_bget?nio:NITINOP_1511) | cutA |
|  | 2-keto-3-deoxy-D-arabino-heptulosonate-7-phosphate synthase I alpha (EC 2.5.1.54) | 2.22 | 0.047 | [NITINOP_1287; NITINOP_2353](http://www.genome.jp/dbget-bin/www_bget?nio:NITINOP_2353) | aroF |
|  | Phosphoribosylformylglycinamidine synthase (EC 6.3.5.3) | 2.22 | 0.047 | [NITINOP_1803](http://www.genome.jp/dbget-bin/www_bget?nio:NITINOP_1800) | purL |
|  | Cytidylate kinase (EC 2.7.4.25) | 2.20 | 0.047 | [NITINOP_1290](http://www.genome.jp/dbget-bin/www_bget?nio:NITINOP_1290) | cmk |
|  | RND efflux system membrane fusion protein (CmeA) | 2.19 | 0.047 | NITNOP_0560; NITNOP_0488; NITNOP_2329 | putative efflux transporter and membrane fusion protein |
|  | Phosphoribosyl-AMP cyclohydrolase  (EC 3.5.4.19) | 2.15 | 0.047 | [NITINOP_0113](http://www.genome.jp/dbget-bin/www_bget?nio:NITINOP_0113) | hisI |
|  | Translation initiation factor 2 | 2.15 | 0.047 | [NITINOP_0786](http://www.genome.jp/dbget-bin/www_bget?nio:NITINOP_0786) | infB |
|  | Integral inner membrane protein of type IV secretion complex | 2.13 | 0.047 | [NITINOP_2980](http://www.genome.jp/dbget-bin/www_bget?nio:NITINOP_2981) | virB |
| No mussels | Lipopolysaccharide heptosyltransferase II | 2.77 | 0.021 | [NITINOP_3124](http://www.genome.jp/dbget-bin/www_bget?nio:NITINOP_3087) | gmhB |
|  | Agmatinase (EC 3.5.3.11) | 2.76 | 0.021 | [NITINOP_1173](http://www.genome.jp/dbget-bin/www_bget?nio:NITINOP_0495) | speB |
|  | NADH-quinone oxidoreductase chain G  (EC 1.6.5.3) | 2.66 | 0.021 | [NITINOP_1374](http://www.genome.jp/dbget-bin/www_bget?nio:NITINOP_1374) | nuoG |
|  | Quinolinate phosphoribosyltransferase; decarboxylating (EC 2.4.2.19) | 2.63 | 0.021 | [NITINOP_2729](http://www.genome.jp/dbget-bin/www_bget?nio:NITINOP_1924) | nadC |
|  | Ribonucleotide reductase of class II coenzyme B12 dependent (EC 1.17.4.1) | 2.57 | 0.021 | [NITINOP_2043](http://www.genome.jp/dbget-bin/www_bget?nio:NITINOP_2043) | nrdJ |
|  | Imidazole glycerol phosphate synthase amidotransferase subunit (EC 2.4.2.-) | 2.51 | 0.021 | [NITINOP_0117](http://www.genome.jp/dbget-bin/www_bget?nio:NITINOP_0117) | hisH |
|  | Pseudouridine synthase family protein | 2.44 | 0.021 | [NITNOP_1829](http://www.genome.jp/dbget-bin/www_bget?nio:NITINOP_0494) | rluB |
|  | Excinuclease ABC subunit C | 2.37 | 0.021 | [NITINOP_1361](http://www.genome.jp/dbget-bin/www_bget?nio:NITINOP_0964) | uvrC |
|  | DNA recombination and repair protein (RecO) | 2.37 | 0.042 | [NITINOP_2378](http://www.genome.jp/dbget-bin/www_bget?nio:NITINOP_2378) | recO |
|  | Heat shock protein | 2.35 | 0.043 | [NITINOP_0609](http://www.genome.jp/dbget-bin/www_bget?nio:NITINOP_2541) | Hsp20 family |
|  | Potassium transporting ATPase (EC 3.6.3.12) | 2.31 | 0.020 | [NITINOP_1503; NITINOP_1504](http://www.genome.jp/dbget-bin/www_bget?nio:NITINOP_1505) | kdpA; kdpB |
|  | DNA polymerase III (EC 2.7.7.7) | 2.29 | 0.042 | [NITINOP_1079](http://www.genome.jp/dbget-bin/www_bget?nio:NITINOP_2678) | dnaX |
